# DNA-Catalyzed Henry Reaction in Pure Water and the Striking Influence of Organic Buffer Systems

**DOI:** 10.3390/molecules20034136

**Published:** 2015-03-04

**Authors:** Marleen Häring, Maria M. Pérez-Madrigal, Dennis Kühbeck, Asja Pettignano, Françoise Quignard, David Díaz Díaz

**Affiliations:** 1 Institute of Organic Chemistry, University of Regensburg, Universitätsstr. 31, Regensburg 93053, Germany; E-Mails: Marleen.Haering@chemie.uni-regensburg.de (M.H.); Maria-del-Mar.Perez-Madrigal@chemie.uni-regensburg.de (M.M.P.-M.); dennis.kuehbeck@gmx.de (D.K.); 2 Institute Charles Gerhardt Montpellier-UMR 5253 CNRS-UMII-ENSCM-UMI, Matériaux Avancés pour la Catalyse et la Santé, 8 rue de l'École Normale, 34296 Montpellier Cedex 5, France; E-Mails: asja.pettignano@enscm.fr (A.P.); francoise.quignard@enscm.fr (F.Q.); 3 IQAC-CSIC, Jordi Girona 18–26, Barcelona 08034, Spain

**Keywords:** DNA, C–C bond formation, nitroaldol reaction, Henry reaction, buffer solutions

## Abstract

In this manuscript we report a critical evaluation of the ability of natural DNA to mediate the nitroaldol (Henry) reaction at physiological temperature in pure water. Under these conditions, no background reaction took place (*i.e.*, control experiment without DNA). Both heteroaromatic aldehydes (e.g., 2-pyridinecarboxaldehyde) and aromatic aldehydes bearing strong or moderate electron-withdrawing groups reacted satisfactorily with nitromethane obeying first order kinetics and affording the corresponding β-nitroalcohols in good yields within 24 h. In contrast, aliphatic aldehydes and aromatic aldehydes having electron-donating groups either did not react or were poorly converted. Moreover, we discovered that a number of metal-free organic buffers efficiently promote the Henry reaction when they were used as reaction media without adding external catalysts. This constitutes an important observation because the influence of organic buffers in chemical processes has been traditionally underestimated.

## 1. Introduction

More than 60 years have passed since the discovery of the structure of deoxyribonucleic acid (DNA)—the molecular basis of life [1]. Beyond DNA technology and its major impact on the pharmaceutical industry, medicine, agriculture and crime scene investigations [2,3,4], considerable research interest has focused on the use of DNA for the fabrication of catalytic systems for organic synthesis [5,6,7]. Within this context, advances in DNA-templated organic synthesis (DTS)—a versatile method for controlling molecular reactivity by modulated effective molarities [8,9]—have generated a large volume of literature. More recently, Chandra and Silverman have reported the catalysis of Diels-Alder reactions by modified DNA-based enzymes [10]. Moreover, DNA-based hybrid materials for application in asymmetric catalysis have been developed by supramolecular or covalent ligation of DNA with a metal complex bearing a specific ligand [11,12,13,14,15]. However, the potential catalytic role of unmodified DNA in organic reactions has only been scarcely investigated. These studies revealed that natural DNA facilitates nitroaldol (Henry) reactions [16] and Michael additions [17,18] in aqueous solutions.

During one of our research programs devoted to investigate the intrinsic catalytic role of biopolymers in C–C bond forming reactions under different conditions [19,20,21,22], we discovered that some buffer aqueous solutions promote the nitroaldol reaction under mild conditions and in high yields. We believe this is a very important observation due to the fact that buffered solutions are traditionally considered as inert dissolution media with few exceptions [23]. However, many biological buffers are based on dissociation equilibriums of organic molecules bearing different functional groups and, therefore, they could influence other chemical transformations. Consequently, we also decided to revisit the course of the nitroaldol reaction in the presence of natural DNA, but in non-buffered water in order to provide accurate information regarding the possible catalytic properties of the DNA.

## 2. Results and Discussion

We chose the reaction between 4-nitrobenzaldehyde (**1a**, 0.1 mmol) and nitromethane (**2a**, 1.9 mmol) at physiological temperature as a model nitroaldol reaction to study the possible background reaction in different solvents (*i.e.*, control experiments in the absence of catalyst) before evaluating the effect of DNA (Table 1). To avoid any loss of product during work-up operations, the crude of the reaction mixtures were strictly analyzed by NMR spectroscopy using a suitable internal standard (see Experimental Section). In good agreement with our previous studies [19,20,21,22], formation of the desired β-nitroalcohol **3a** was not observed in organic solvents such as THF, toluene, DMSO or EtOH (entry 1). However, **3a** was formed in high yields when 20 mM buffers MES (pH 5.5), MOPS (pH 6.5), TRIS (pH 7.5) or HEPES (pH 7.5) were used as solvent (entries 2–5). Amongst these buffers, MOPS and TRIS were the most effective, affording **3a** in *ca*. 85% yield at 37 °C within 8 h. In sharp contrast, only traces (≤4%) of **3a** were detected in distilled water (pH 6.5) (entry 6). It is worth mentioning that there are several recipes for preparing a buffering medium, including the addition of different metal salts (e.g., NaCl, MgCl_2_) especially for biochemical reactions [24]. Although preliminary experiments did not show major differences in the reaction outcome caused by the presence of those salts (entries 7 and 8), we decided to run the experiments in buffers without metal ions in order to completely exclude any background participation in the catalysis of the nitroaldol reaction (entry 7). In order to avoid any shift in dissociation, we also kept constant the concentration of the buffers and we prepared them at the same temperature at which we planned to perform the reactions (37 °C). In any event, the change of the corresponding p*K*_a_ values of the buffers used in this work is very small (∆p*K*_a_ ~0.2–0.5) when prepared at the same concentration within the range of temperature between 20 °C and 37 °C. Therefore, no major kinetics differences associated to the pH of the medium can be expected during the nitroaldol reaction in buffered solutions if temperature fluctuations occur within the mentioned range. These effects may be predicted using online calculators [25].

**Table 1:** Effect of solvent in the model nitroaldol reaction between **1a** and **2a**. ^a^

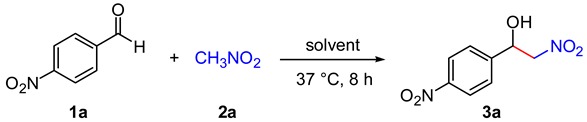

Entry	Solvent System	Yield ^e^ of 3a (%)
1	DMSO, EtOH, THF, toluene	0 ^f^
2	MES (pH 5.5)	70
3	MOPS (pH 6.5)	86
4	HEPES (pH 7.5)	71
5	TRIS (pH 7.5)	83
6	H_2_O (pH 6.5) ^b^	≤2 (4) ^g^
7	H_2_O + MgCl_2_ (pH 6.5) ^c^	5
8	H_2_O + NaCl (pH 6.5) ^d^	≤2

^a^ Reaction conditions: **1a** (0.1 mmol), **2a** (1.9 mmol), solvent (0.5 mL), 37 °C, 8 h. No significant change in the pH of the medium was observed during the reaction (∆pH < 0.1); ^b^ Medium’s intrinsic pH. Reaction carried out without the adjustment of pH; ^c^ Reaction performed in the presence of 50 mM MgCl_2_; ^d^ Reaction carried out in the presence of 150 mM NaCl; ^e^ ^1^H-NMR yields that correspond to the average values of at least three independent experiments; ^f^ Formation of undesired side products was detected only in the case of EtOH. No attempts were made to separate and characterize these products; ^g^ Yield after 24 h.

Different aldehydes were also tested in the nitroaldol reaction with nitromethane (**2a**) in MOPS to ensure that the observed buffer effect was not specific of 4-nitrobenzaldehyde (**1a**) (Table 2). Not surprisingly, electron-poor aromatic or heteroaromatic aldehydes such as 2-pyridinecarboxaldehyde (**1c**) were converted to the nitroaldol product **3** more efficiently than electron-rich aromatic aldehydes such as 4-chlorobenzaldehyde (**1d**).

**Figure 1:**
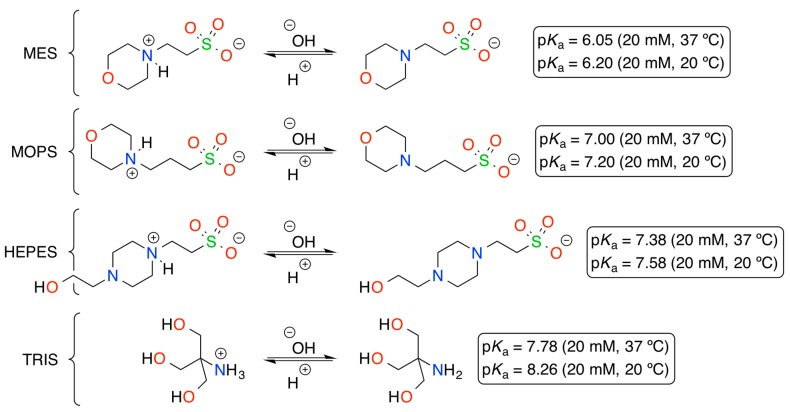
Dissociation steps and pK_a_ values at 25 °C and 37 °C of the buffers used in this work (values estimated at 20 mM concentration) [26]. Buffer names: MES = 2-(N-morpholino) ethanesulfonic acid; MOPS = 3-(N-morpholino)propanesulfonic acid; HEPES = 4-(2-hydroxyethyl)-1-piperazineethanesulfonic acid; TRIS = tris(hydroxymethyl)aminomethane.

The foregoing results point out that organic buffers are not inert and can interact with reactants during a chemical process [26]. Contribution of these substances in aldol-like reactions with active methylene compounds is not totally unexpected if we take into consideration the chemical structures of the buffers bearing basic centers and their dissociation constants (Figure 1), as well as different mechanisms of the nitroaldol reaction under varied conditions (e.g., base-iminium catalysis, Brønsted-Lowry acid-base dual catalysis) [27,28,29,30]. Thus, the existing equilibrium between the weak acid and its conjugate base in buffer solutions makes possible their participation (at least a small molar fraction) in other reversible processes (e.g., generation of *aci*-nitro intermediate via the formation of a complex between buffer and nitroalkane) with continuous pH restoration according to Le Chatelier’s principle.

**Table 2:** Nitroaldol reaction in MOPS buffer between different aldehydes **1** and **2a**. ^a^

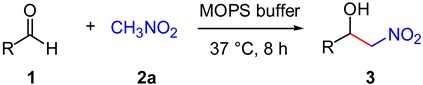

Entry	R	1	Yield ^b^ of 3 (%)
1	(4-NO_2_)-C_6_H_4_	**1a**	86
2	(2-NO_2_)-C_6_H_4_	**1b**	87
3	Pyrid-2-yl	**1c**	90
4	(4-Cl)-C_6_H_4_	**1d**	21
5	Furfur-2-yl	**1e**	27

^a^ Reaction conditions: **1** (0.1 mmol), **2a** (1.9 mmol), MOPS buffer (0.5 mL, pH 6.5), 37 °C, 24 h; ^b^ ^1^H-NMR yields that correspond to the average values of at least two independent experiments

With these results in hand, we decided to use non-buffered purified water as solvent to unequivocally establish the potential catalytic role of DNA in the nitroaldol reaction at physiological temperature. Preliminary screening of the reactions conditions (see Experimental Section, Table 4) showed that 2 mg of double-stranded DNA-sodium salt from salmon sperm (ssDNA) and tenfold molar excess of **2a** with respect to **1a** were optimal to obtain the corresponding β-nitroalcohol **3a** in good yield (80%) within 24 h. Further increase of the reaction time to 48 h enhanced the yield to 93% but also the background reaction (*i.e.*, control without ssDNA) to 15%. This is an important observation because the use of even longer reaction times may increase considerably the background reaction. Higher loadings of ssDNA did not significantly improve the yield either. On the other hand, pure samples of sodium-free single-stranded ssDNA behaved similar to double-stranded ssDNA-sodium salt. No major differences where observed with pure DNA samples obtained from different sources (*i.e.*, salmon sperm, calf thymus). Interestingly, DNA from herring sperm (hsDNA) was totally inactive in the model reaction even at higher loading. While the reason for this remains unclear, the large differences in the number of base pairs between both DNA samples (ssDNA ~2000 bp; hsDNA ~50 bp) and partial degradation of commercial hsDNA sample may be in part responsible.

Although deoxyribose sugar molecules existing in the DNA are unlikely to participate in the nitroaldol reaction, the nitrogeneous nucleobases (%G-C content of DNA from salmon testes is 41.2%) could be involved in this base-catalyzed reaction. Moreover, the phosphate groups presented in the structure of the biomolecule could also provide a catalytic synergistic effect to the nucleobases. However, further research using different sequences of DNA samples is required to fully understand the specific catalytic centers of DNA involved in this process.

At this point, different aldehydes were tested using the optimized conditions in order to ascertain the substrate scope of the reaction (Table 3). Aromatic aldehydes bearing strong or moderate electron-withdrawing groups satisfactorily reacted with nitromethane (**2a**) affording the β-nitroalcohols **3** in good yields within 24 h (entries 1, 3–4 and 12). Possible byproducts such as dehydrated (nitroalkenes) or 1,3-dinitro derivatives were only observed at higher temperatures (*vide infra*). The screening also showed the influence of the substitution position on the product yield, leading to a significant decrease with the *ortho*- and *meta*-substituted aldehydes in comparison to the *para*-substituted isomer (entries 3 and 4 *vs.* entry 1). Heteroaromatic aldehydes such as 2-pyridine-carboxaldehyde (entry 6) and furfural (entry 9) were also converted into the corresponding β-nitroalcohols **3** in good and modest yields, respectively. However, aliphatic aldehydes (e.g., isovaleraldehyde) remained unreacted under these conditions, whereas low yields were obtained with benzaldehyde (entry 10), 4-chlorobenzaldehyde (entry 8) or electron-rich aromatic aldehydes (e.g., entry 11) even after longer periods of time. While being out of the scope of this manuscript, the use of phase transfer co-catalysts (e.g., CTAB) could be also considered in order to improve these yields, at least to a certain extent [31].

On the other hand, we found that the yields dropped significantly when nitroethane (**2b**) was used as donor instead of nitromethane (**2a**) (entries 2, 5 and 7 *vs.* entries 1, 4 and 6, respectively). Besides the higher water-solubility of **2a** *vs.* **2b** [32], these results also suggest that the size of the carbanion plays a more important role than the p*K*_a_ of the donor (p*K*_a_ (**2a**) = 10.2; p*K*_a_ (**2b**) = 8.6 [33]) in this process. Moreover, slight *syn*-diastereoselectivities were found in these examples. A water-assisted cyclic chair-like transition state, formed by hydrogen-bonding between the oxygen atom of the aldehyde and the hydrogen atom of the *aci*-nitro tautomer of nitroethane, has been recently proposed to explain preferential formation of *syn* nitroaldol products in phosphate buffer [31].

**Table 3:** ssDNA-catalyzed nitroaldol reaction. ^a^

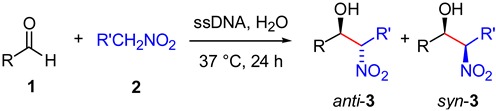

Entry	R (1)	1	R'	2	Yield ^b^ of 3 (%)	dr ^d^ (*anti*/*syn*)
1	(4-NO_2_)-C_6_H_4_	**1a**	H	**2a**	80	-
2	(4-NO_2_)-C_6_H_4_	**1a**	CH_3_	**2b**	18 (46) ^c^	1:1.7
3	(3-NO_2_)-C_6_H_4_	**1f**	H	**2a**	57	-
4	(2-NO_2_)-C_6_H_4_	**1b**	H	**2a**	51	-
5	(2-NO_2_)-C_6_H_4_	**1b**	CH_3_	**2b**	12	1:2.1
6	Pyrid-2-yl	**1c**	H	**2a**	84	-
7	Pyrid-2-yl	**1c**	CH_3_	**2b**	64	1:1.3
8	(4-Cl)-C_6_H_4_	**1d**	H	**2a**	19 ^c^	-
9	Furfur-2-yl	**1e**	H	**2a**	37 ^c^	-
10	C_6_H_5_	**1g**	H	**2a**	4 ^c^	-
11	(4-OH)-C_6_H_4_	**1h**	H	**2a**	6 ^c^	-
12	(4-NC)-C_6_H_4_	**1i**	H	**2a**	61	-

^a^ Reaction conditions: **1** (0.1 mmol), **2** (1.0 mmol), ssDNA (2 mg), H_2_O (0.5 mL), 37 °C, 24 h. No significant change in the pH of the medium was observed during the reaction (∆pH < 0.1); ^b^ ^1^H-NMR yields that correspond to the average values of at least two independent experiments; ^c^ Yield after 48 h; ^d^ Diastereomeric ratio *anti/syn* determined by ^1^H-NMR analyses.

While the DNA could be reused for several runs in the nitroaldol reaction, a gradual deactivation of the catalyst was observed after each run (e.g., the yield of the model reaction between **1a** and **2a** (Table 3, entry 1) dropped to *ca*. 30% in the 4th run). Such activity lost has been also described with other catalytic biopolymers, where factors such as the evolution of intermediate imines (e.g., formation of crosslinked aminals, reduction via Cannizaro-type reactions), the use of protic solvents and large excess of donor, as well as the slow reaction kinetics, could contribute to block the basic catalytic sites [17,18]. In this sense, an inefficient molecular desorption from the catalyst seems to be a critical issue in biopolymer-catalyzed nitroaldol reactions, which require future studies in this direction.

Despite the unique chiral structure of the DNA, chiral HPLC of the reaction mixtures revealed negligible enantioselectivity during the nitroaldol reaction at 37 °C in pure water. This poor enantioselectivity has been also reported for other catalytic biopolymers in aldol-like reactions [19,20,21,22,34,35,36,37]. However, slight enantioselectivity (*ca*. 20% ee) could be observed when DMSO was used as solvent.

**Figure 2:**
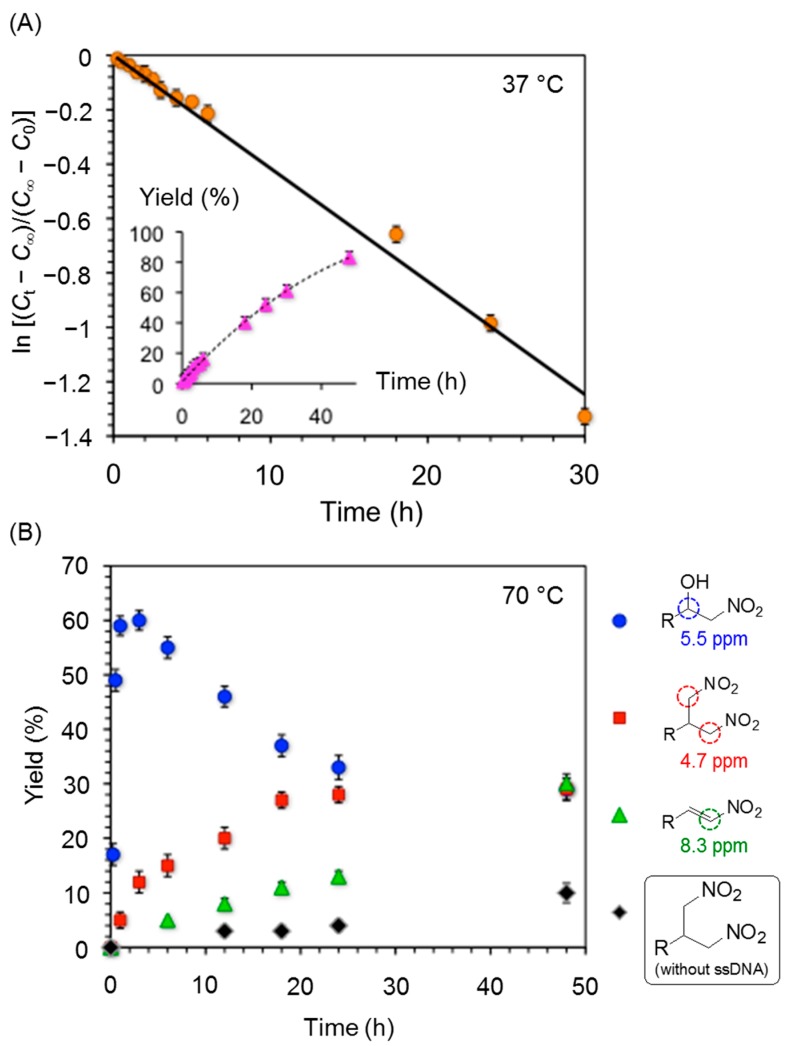
(**A**) First-order kinetics plot of the model nitroaldol reaction between **1a** and **2a** catalyzed by ssDNA as described in Table 3. C_∞_ = final concentration, at infinite time; C_t_ = concentration at given time t; C_0_ = initial concentration, at t = zero time. R^2^ = 0.99. Inset: Evolution of reaction conversion over time; (**B**) Formation of β-nitroalcohol **3a**, nitroalkene and 1,3-dinitro derivatives from the reaction between **1a** (0.1 mmol) and **2a** (1.0 mmol) catalyzed by ssDNA (2 mg) at 70 °C. Kinetics profile of the 1,3-dinitro compound in the absence of ssDNA is also shown.

The first-order kinetic analysis of the model reaction between **1a** and **2** established a slow rate constant of *k* = (4.2 ± 0.5) × 10^−2^ h^−1^ (Figure 2A). In addition, and keeping in mind recent reports dealing with 1,4-Michael additions catalyzed by DNA in aqueous media [17,18], we explored the possibility to perform a nitroaldol-elimination-Michael tandem reaction using acetophenone or cyclohexanone as Michael donor. Unfortunately, the high temperatures (*ca*. 70 °C) required for both the formation of the nitroalkene intermediate and the subsequent Michael reaction caused the competitive formation of undesired side products such as 1,3-dinitro derivatives (Figure 2B), making the tandem process too impractical under these conditions. Interestingly, control kinetics studies revealed that ssDNA also catalyzes the formation of the 1,3-dinitro derivative at 70 °C. For instance, only traces (< 5%) of this byproduct could be detected when the model mixture **1a** + **2a** was heated at 70 °C in pure water for 24 h. However, the 1,3-dinitro compound was formed in *ca*. 30% when the experiment was repeated in the presence of ssDNA. Further investigations are in progress in this direction.

## 3. Experimental Section

### 3.1. Materials

Unless otherwise indicated, analytical grade solvents and reactants were commercially available and used as received without further purification. Aldehydes (purity by GC > 98%) were mainly purchased from TCI Europe. Milli-Q water and freshly buffer solutions were used for the experiments in aqueous solutions. Pure double-stranded DNA sodium salt from salmon sperm (ssDNA; 41.2% G-C content; molecular mass 1.3 × 10^6^ Da; *ca*. 2000 bp; CAS 68938-01-2; Cat. No. D1626), sodium-free single-stranded DNA from salmon sperm (CAS 9007-49-2; Cat. No. D7656), DNA from herring sperm (hsDNA; partially degraded; <50 bp; CAS 68938-01-2; Cat. No. D3159) and DNA sodium salt from calf thymus (CAS 73049-39-5; Cat. No. D1501) were supplied by Sigma-Aldrich. Buffers HEPES (CAS 7365-45-9; Cat. No. H3375), TRIS (CAS 77-86-1; Cat. Nr. T1503), MES (CAS 4432-31-9; Cat. Nr. M3671) and MOPS (CAS 1132-61-2; Cat. Nr. M1254) were purchased from Sigma-Aldrich.

### 3.2. Methods

^1^H-NMR spectra were recorded on Avance 300 or Avance 400 spectrometers (Bruker) at 25 °C. Chemical shifts for ^1^H-NMR were reported as δ, parts per million, relative to external standards. Yields and diastereomeric ratios (*anti/syn*) were determined by ^1^H-NMR analyses of the crude product in CDCl_3_ using diphenylmethane (0.1 mmol, 16.7 µL), dimethyl acetamide (0.1 mmol, 9.2 µL) or 1,3,5-trimethoxybenzene (0.1 mmol, 16.8 mg) as internal standard after complete work-up of the reaction. Relative configurations were assigned by comparison with ^1^H-NMR data reported in the literature [38,39]. For instance, in the model reaction between **1a** and **2b**, the *anti* diastereomer was identified by a doublet at 4.85 ppm (*J* = 8.3 Hz), whereas the *syn* diastereomer displayed the doublet at 5.41 ppm (*J* = 2.4 Hz). For kinetics calculations, the ^1^H-NMR analyses of the reaction mixtures were performed in the presence of an internal standard as above indicated. In general, given yield values correspond to the average of at least two independent measurements with STDV ±2%–4%. Among various kinetics models, the straight lines shown in the kinetics plots correspond to the best fit of the first-order model (e.g., [nitromethane] ≥ [aldehyde]). Analytical thin layer chromatography (TLC) was performed on fluorescent-indicating plates (aluminium sheets precoated with silica gel 60 F254, Merck). Reactions were monitored by TLC and visualized by the use of the phosphomolybdic acid as stain solution and UV light (254 nm). Column flash chromatography was performed using Merck silica gel (70–230 mesh) from Merck or silica gel (100–200 mesh). Mass spectra were recorded at the Central Analytical Laboratory at the Department of Chemistry of the University of Regensburg on a Varian MAT 311A (Palo Alto, CA, USA), Finnigan MAT 95 (Bremen, Germany), Thermoquest Finnigan TSQ 7000 or Agilent Technologies 6540 UHD Accurate-Mass Q-TOF LC/MS (Agilent Technologies, Santa Clara, CA, USA). High performance liquid chromatography was carried out on a HPLC 335 detector on a 325 system by Contron Instruments (Fairfield, NJ, USA). Phenomena Lux Cellulose-1, 4.6 mm × 250 mm, 5 µm column (eluent: *n*-heptane-*i*-PrOH 70:30; flow 1.0 mL/min; λ = 254 nm) was used to determine potential enantioselectivity.

### 3.3. Preliminary Optimization Experiments

The model reaction between **1a** and **2a** was carried out at 37 °C under different experimental conditions of solvent, reaction time and catalyst loading. Table 4 shows a selection of the most relevant experiments.

**Table 4:** Initial screening of reaction conditions. ^a^

Entry	Catalyst	Solvent	Time (h)	Catalyst Loading (mg)	Conditions	Yield ^c^ of 3a (%)
1	hsDNA	H_2_O	8	10	A	0
2	ssDNA	H_2_O	8	10	A	25
3	ssDNA	H_2_O	24	10	A	38
4	ssDNA	H_2_O	48	10	A	77
5	ssDNA	H_2_O	24	2	B	80
6	ssDNA	H_2_O	48	2	B	93
7	hsDNA	H_2_O	24	40	B	0
8	Na^+^-free ssDNA	H_2_O	24	2	B	77
9	-	H_2_O	24	-	B	4
10	-	H_2_O	48	-	B	15
11	-	DMSO	24	-	B	31
12	ctDNA ^b^	DMSO	24	2	B	76

^a^ Reaction conditions: (A) **1a** (0.5 mmol), **2a** (9.5 mmol), DNA (2 mg), solvent (3 mL), 37 °C. (B) **1a** (0.1 mmol), **2a** (1.0 mmol), DNA (2 mg), solvent (0.5 mL), 37 °C. Note that studies at other temperatures were not performed in this project. Both selectivity and kinetics data could be altered at different temperatures; ^b^ DNA sodium salt from calf thymus; ^c^ ^1^H-NMR yields obtained from the average of at least two independent experiments.

### 3.4. General Procedure for ssDNA-catalyzed Henry Reaction

ssDNA (2 mg) was added in one portion to a mixture of 4-nitrobenzaldehyde (**1a**, 0.1 mmol, 15.1 mg), nitromethane (**2a**, 1.9 mmol, 103 µL) and solvent (0.5 mL) placed into a screw cap vial (4 mL). The resulting reaction mixture was gently stirred (250 rpm) at 37 °C for 24 h. After completion, water (1 mL) was added. The reaction mixture was washed with EtOAc (3 × 5 mL), dried over anhydrous sodium sulfate, filtrated and evaporated under reduced pressure. To determine the NMR yield, the obtained crude product was dissolved in CDCl_3_ and the internal standard (*vide supra*) added to the solution. Note: The same procedure, albeit in the absence of ssDNA, was used for studying the effect of the buffers on the reaction.

### 3.5. Typical Recycling Procedure

After the reaction, the aqueous phase was washed with EtOAc (4 × 3 mL) in the reaction vial. After removal of the organic phase, the vial with the aqueous phase was subjected to N_2_-flow prior addition of the reaction substrates for the next run.

## 4. Conclusions

In conclusion, ssDNA is able to catalyze the reaction between electron-poor aromatic aldehydes or heteroaromatic aldehydes and nitroalkanes at physiological temperature in pure water. The process is governed by a first-order kinetics and the corresponding β-nitroalcohols are selectively formed within 24 h in good yields. The intrinsic catalytic activity of DNA should be therefore taken into account during the development of DNA-hybrid catalysts. Moreover, this investigation demonstrated that metal-free organic buffers (e.g., MES, pH 5.5; MOPS, pH 6.5; HEPES, pH 7.5; TRIS, pH 7.5) also efficiently promote the nitroaldol reaction when they are used as reaction media in the absence of external catalysts. Finally, we would like to stress that studying the potential of biopolymers to promote selective formation of C-C bonds (a prerequisite for all life in earth), even when they performance is usually inferior to standard catalysts, could bring important insights into the molecular mechanism underlying evolution, and also help for the design of safer and “greener” catalysts in the future. It is under this perspective where our research program acquires higher relevance.
